# Prospective Comparison of 24-Hour Urine Creatinine Clearance with Estimated Glomerular Filtration Rates in Chronic Renal Disease Patients of African Descent

**DOI:** 10.3390/medicines8090048

**Published:** 2021-09-01

**Authors:** Marlene Tapper, Donovan A. McGrowder, Lowell Dilworth, Adedamola Soyibo

**Affiliations:** 1 Department of Pathology, Faculty of Medical Sciences, The University of the West Indies, Kingston 7, Jamaica; teeanmarl@gmail.com (M.T.); lowell.dilworth02@uwimona.edu.jm (L.D.); 2 Department of Medicine, Faculty of Medical Sciences, The University of the West Indies, Kingston 7, Jamaica; drsoyibo@gmail.com

**Keywords:** glomerular, filtration, rate, creatinine, estimate, cystatin C, kidney, disease, chronic

## Abstract

Background: The 24-hour (24-h) creatinine clearance (CrCl) is the most common method for measuring GFR in clinical laboratories. However, the limitations of CrCl have resulted in the widespread acceptance of mathematically derived estimated glomerular filtration rate (eGFR) using Cockcroft-Gault (CG), Modification of Diet in Renal Disease (MDRD) and the Chronic Kidney Disease Epidemiology Collaboration (CKD-EPI) equations in predicting eGFR. The aim of the study was to compare 24-h CrCl with eGFR derived from these formulae and to identify which could be the best alternative. Method: A prospective study was conducted involving 140 CKD patients. Creatinine and cystatin C concentrations were determined using the cobas 6000 analyzer. The eGFR was calculated using the CG formula, 4-variable MDRD and CKD-EPI equations, and Bland-Alman plots bias was determined. Results: The CG and MDRD formulas had mean eGFR values similar to CrCl and correlation coefficients (r) were highest for CG (0.906) and lowest for MDRD (0.799). The CG equation was in agreement with 24-h CrCl in all but stage V CKD while the MDRD equation compared well in all except Stage IV CKD. The CG equation was positively biased (0.9857) while the MDRD had a negative bias (−0.05). Conclusion: The Cockcroft-Gault formula provides a more accurate assessment of GFR than 24-h CrCl and would be recommended as a substitute to provide the best estimate of GFR in our population.

## 1. Introduction

Worldwide, chronic kidney disease (CKD) has been acknowledged as a public health challenge and over the last decade there has being significant increase in both incidence and prevalence in various populations [1]. In 2017, GBD Chronic Kidney Disease Collaboration reported that the global mortality of CKD persons was 1.2 million, and the worldwide death rate was 41.5% greater in 2017 compared with 1990 [2]. In the same report there was a worldwide prevalence of 9.1% representing 697.5 million cases [2]. In a systematic and meta-analysis of 100 observational studies consisting of approximately 6.9 million patients, the CKD was 13.4% for stage 1–5 and 10.6% for stage 3–5 [3]. In general, there is an age-associated decrease in renal function in CKD that is accelerated in diabetes mellitus and hypertension (the most common causes) and also primary renal disorders such as glomerulonephritis and interstitial nephritis [4]. Data from the Caribbean Renal Registry showed that diabetes mellitus, hypertension and chronic glomerulonephritis were the major causes of CKD and end stage renal disease (ESRD) in English-speaking Caribbean countries [5,6].

The glomerular filtration rate (GFR) provides a precise measure of renal function and according to Kidney Disease Quality Outcome Initiative, CKD is defined by GFR < 60 mL/min/1.73 m^2^ due to kidney injury or lessened function that persist for three months or more [1]. Furthermore, CKD is also established by the incidence of albuminuria which is defined as spot urine albumin/creatinine ratio more than 30 mg/g in at least two of three spot samples [7]. On occasions where a 24-h urine sample was collected from the patient, the presence of microalbuminuria in defining CKD is substantiated by an albumin excretion rate ≥ 30 mg/24 h [8].

Kidney function can be assessed by the determination of measured GFR (mGFR) via the clearance from the plasma of administered exogenous filtration substances such as inulin, the gold standard as well as iohexol [9]. Renal inulin clearance provides the most accurate assessment of mGFR but it is compromised by its high cost, invasiveness, complex procedure requiring multiple infusions, challenges of collecting timed catheterized urine samples and lack of inulin availability. Therefore, renal inulin clearance is impractical in routine clinical practice [10]. The urinary clearance of radioisotopes or radiotracer molecules such as ^125^I-iothalamate, technetium-99m diethylenetriamine-pentaacetic acid (^99m^Tc-DTPA) or chromium-51 (^51^Cr)EDTA in the determination of mGFR, have been found to be reliable and precise in patients with ESRD [11], but the use of the single injection technique may overestimate mGFR in individuals with normal kidney function [12].

Creatinine clearance (CrCl) is a mGFR methodology that provides an assessment of renal function based on the clearance of creatinine from a given volume of blood plasma per unit time. CrCl involves the determination of creatinine in plasma and an accurately timed 24-h urine sample, and the biomarker satisfy most of the criteria as an ideal marker for mGFR assessment including being present in fairly stable concentration in the blood and freely filtered at the glomerulus [13]. However, the accuracy of the mGFR determined by CrCl is compromised by incomplete urine collection and inconvenience to patients, and systematic overestimation of GFR by a marginal error of about 10–20% as a result of age-related tubular secretion from the peritubular capillaries [14]. Furthermore, CrCl is affected by gender, ethnicity, dietary intake, muscle wasting disorders, and intra- and inter-viability among persons [15]. Despite these concerns, CrCl, it is regarded as a fast, cost-effective and a practical method for determining mGFR and all efforts should be made to reduce potential errors [16].

Estimated GFR (eGFR) equations such as the Cockcroft-Gault (CG) and the Modification of Diet in Renal Disease (MDRD) formulas were recommended by The National Kidney Foundation Dialysis Outcome Quality Initiative (K/DOQI) in the assessment of kidney function particularly in individuals with acute or chronic renal disease [17]. The CG formula reported in 1976 was established using serum creatinine to predict CrCl in 236 adult hospitalized patients in the absence of a 24-h urine collection [18]. The CG formula uses a patient’s gender and weight to predict GFR and have been applied to non-diabetic patients with ESRD where there was an overestimation when compared with inulin clearance [19].

The original MDRD equation reported in 1999 was developed to predict GFR using serum creatinine levels and other factors such as age, gender and ethnicity. The equation was assessed in a cross-sectional study comprising 1628 persons with variable degrees on kidney impairment using a stepwise regression procedure, with standard GFR determined from renal clearance of ^125^I-iothalamate [20]. Simplified versions of the MDRD formula using five and four variables to predict GFR were proposed using the same cohort of 1628 patients and found to have equivalent performance to the original 6-variable formula and more accurate estimates than CG and CrCl [21]. There are other studies including a large European cohort of 2095 adult Europeans, and another consisting of 828 CKD patients that reported better predictive performance of the MDRD than CG formula because of greater precision and lower bias [22,23]. However, while these studies provides more evidence of the validation of the MDRD formula in patients with moderate and severe renal disease, it is limited by systematic bias in individuals with mildly decreased or normal GFR [23].

The Chronic Kidney Disease Epidemiology Collaboration (CKD-EPI) formula was established in 2009 and validation data showed that it performed better than the MDRD formula particularly at higher GFR with greater accuracy, less bias and better precision [24]. There are serum creatinine (crea) and cystatin C (cys)-based CKD-EPI equations, and clinical trials have presented validation data and compared their clinical performance in accurately predicting GFR [25,26,27]. The CKD-EPI equations include CKD-EPI_crea_, CKD-EPI_cys_ and CKD-EPI_crea/cys_, and a recent meta-analysis comprising 35 studies with 23,667 participants found that estimates of CKD-EPI_cys_ and CKD-EPI_crea/cys_ were closer to mGFR and were more accurate and less bias compared with CKD-EPI_crea_ [28].

In this study, the eGFR formulas are considered as alternatives to 24-h CrCl and in the latter the collection of urine is cumbersome with the possibly introduction of errors. Therefore, the eGFR formulas are proposed as tools to circumvent the urine collection with possibly improved overall eGFR results.

Nevertheless, the most common routine and practical method for measuring GFR is accomplished with a 24-h urine collection for CrCl evaluation. The aim of the study was to compare 24-h CrCl with eGFR using the formulas (MDRD, CG, CKD-EPIcrea, CKD-EPIcys and CKD-EPIcrea/cys) and to identify which amongst the eGFR formulas closely correlates with 24-h CrCl. The study also sought to (i) evaluate how well eGFR values stratifies patients with CKD, (ii) determine the correlation of eGFR to 24-h CrCl and (iii) examine the mean values of the different formulas to stage of CKD.

## 2. Materials and Methods

### 2.1. Patient Recruitment and Ethical Approval

A description of the study was previously published [29]. In summary, this is a prospective study where patients were recruited from the renal clinic at the University Hospital of the West Indies (UHWI) between February 2016 and May 2016. In this period, 140 patients from 18 to 97 years of age were recruited. The study included cases from all Jamaica encompassing those from western parishes. Patients agreed to participate in the study were assigned data entry numbers in order to maintain confidentiality [29].

The study received approval from The University of the West Indies/University Hospital of the West Indies Faculty of Medical Sciences Ethics Committee and the protocol for the conduct of research outlined were adhered to.

The inclusion criteria for persons recruited to the study were as follows: aged 18 to 98 years; CKD with reduced renal function (stage 1 to 4); aetiologies of chronic disease such as diabetes mellitus (type 1 and 2), hypertension, systemic lupus erythematosus, obstructive uropathy, chronic glomerular nephritis and autosomal dominant polycystic kidney disease, and compliance with instructions concerning urine sample collection.

The exclusion criteria for persons recruited to the study were as follows: aged less than 18 years; normal renal function; multiple myeloma and cancers such as prostate, breast and colorectal; current use of immunosuppression, previous kidney transplantation; and severe comorbid disorders such as chronic active hepatic, cirrhosis and congestive cardiac failure.

### 2.2. Demographic Data Collection

Demographic data, including age, gender, date of birth, height and weight were collected. Height and weight were measured in the renal clinic. The date of CKD diagnosis, age, stage and cause of renal impairment at diagnosis as well as any comorbid conditions were ascertained from the patients themselves and/or confirmed by docket search [29].

### 2.3. Measurements

The footwear of the patients were removed and their height measured to the nearest 0.5 cm by means of a wooden platform with height rule. The weight of the patients was measured to the nearest 0.5 kg with manual Seca 761 scales (Vogel & Halke, Hamburg, Germany) after removed of footwear. Having determined the weight and height the body mass index (BMI) was calculated as weight in kilograms divided by height in metres squared (kg/m^2^).

### 2.4. Blood Samples and 24-Hour Urine Collections

Seven (7) mL of venous blood was obtained from each participant by venipuncture from the antecubital fossa or another convenient site. The samples were collected in Vacutainer tubes and allowed to clot for 30 min. Separation was carried out by centrifugation at 3500 rpm for 5 min at room temperature and then the serum was aliquoted.

Blood samples were processed within three hours of receipt in the Chemical Pathology Laboratory at the Department of Pathology, The University of the West Indies. Specimens were stored at −70 °C for assays not completed within 24-h.

At the time of consent, the participants were given directives on the proper collection of a 24-h urine sample. They were asked to repeat the instructions to ensure comprehension [29]. Participants were advised to empty their bladder and record the time of voiding.

The 24-h urine specimen was submitted on the morning of completion of collection. To determine completeness patients were asked to describe the process followed and volume was also deemed adequate based on normal excretion rate (males 20–25 mL/kg/day and females 15–20 mL/kg/day).

Each participants provided two consecutive 24-h urine collections (in a 4.5 litre plastic bottle containing thymol as the preservative). The volume of urine in each bottle was determined and 55-mL aliquots of urine were pipetted into test tubes and frozen at −20°C until analyzed for creatinine.

### 2.5. Serum Assays Used to Determine Analytes

Serum and urine biochemistry tests were performed on the cobas 6000 (Roche/Hitachi, Roche Diagnostics, Indianapolis, IN, USA) analyser [29]. Serum and urine creatinine were measured using a Jaffe alkaline picrate method, a kinetic colorimetric assay (Roche Diagnostics). This method of creatinine determination has been validated against isotope dilution mass spectrometry (IDMS) standards [30]. “Rate-blanking” limits interference by bilirubin and the results are adjusted by −26 μmol/L to correct for pseudochromogens [31].

For urine creatinine a 1:10 dilution was automatically performed by the c501 module of the cobas 6000 analyzer before the assay is initiated. On reaction with picric acid at alkaline pH a yellow-orange complex is produced. The development of color being equivalent to the concentration of creatinine in the urine as well as serum samples [32].

Cystatin C was measured by the Tina-quant Cystatin C Gen. 2, a particle enhanced immuno-turbidimetric assay that is standardized against ERM-DA471/IFCC (The International Federation for Clinical Chemistry and Laboratory Medicine) reference material. Cystatin C in the specimen binds to anti-cystatin C-coated latex particles and the degree of turbidity from the reaction is measured at 546 nm, and is equivalent to cystatin C concentration [33].

### 2.6. Creatinine Clearance and Estimated GFR

The CrCl (mL/min/1.73 m^2^) from the 24-h urine collection was calculated in the laboratory information management system (LIMS), Chemical Pathology Laboratory, Department of Pathology, The University of the West Indies. The eGFR was calculated using the CG formula, 4-variable isotope dilution mass spectrometry (IDMS) traceable MDRD, CKD-EPI_crea_, CKD-EPI_crea/cys_ and CKD-EPI_cys_ equations and reported in mL/min [24,34,35]. Patients were placed into the five stages of CKD by the different methods then assessed against the stage determined by 24-h CrCl.

CKD is defined as eGFR < 60 mL/min/1.73 m^2^. The stages of eGFR were categorized based on the classification system established by the National Kidney Foundation Kidney Disease Outcomes Quality Initiative classification where stage 3 = eGFR of 30 to 59 mL/min/1.73 m^2^, stage4 = eGFR of 15 to 29 mL/min/1.73 m^2^, and stage 5 = eGFR of <15 mL/min/1.73 m^2^. Stage 3 was further classified into 3a (eGFR of 45–59.9 mL/min/1.73 m^2^) and 3b (eGFR of 30–44.9 mL/min/1.73 m^2^) [36].

### 2.7. Data Analysis

The data analysis was conducted using the IBM Statistical Programme of the Social Science (SPSS) version 22 and Microsoft Excel. Demographic characteristics are presented as mean ± standard deviation (SD). The frequency of different causes of CKD were determined. The Pearson coefficient (r) was used to assess the correlation of results by conventional 24-h urine CrCl and (1) CG formula and (2) eGFR by the (i) four-variable IDMS traceable MDRD (ii) CKD- EPI_crea_ (iii) CKD-EPI_cys_ and (iv) CKD-EPI_crea/cys_ formulas. A *p*-value < 0.05 (two- tailed) indicated statistical significance.

Results were also analyzed by linear regression and scatter plots. The Kolmogorov-Smirnov test was used to determine normality and Bland-Alman plots bias. Comparisons of the mean eGFRs at different stages of kidney disease were assessed by the paired t-test at significance level *p* < 0.05 (two-tailed).

## 3. Results

**Figure 1:**
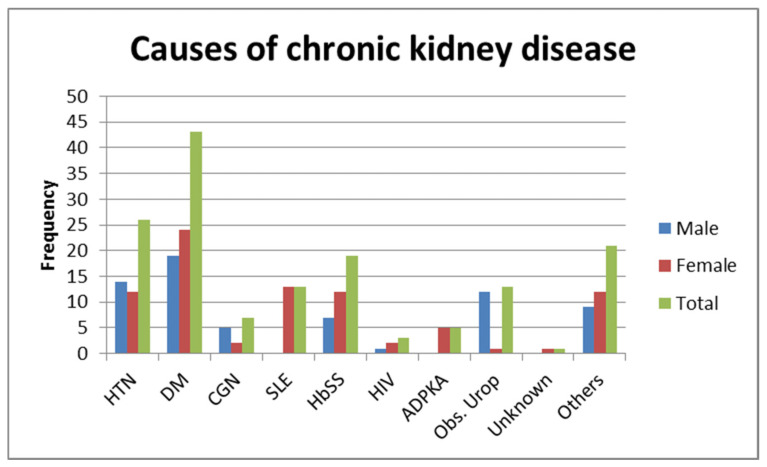
Causes of chronic kidney disease. HTN—hypertension; DM—diabetes mellitus; CGN—chronic glomerular nephritis; SLE—systemic lupus erythematosus; HbSS—haemoglobin SS; HIV—human immunodeficiency virus; ADPKA—autosomal dominant polycystic kidney disease; Obs. Urop.—Obstructive uropathy.

The etiologies of CKD in this group of patients was previously published [27]. The majority of the patients with CKD presented with diabetes mellitus followed by hypertension (Figure 1). The majority of patients, 64 (45.7%), had Stage III disease at diagnosis. There were 5 patients undergoing renal replacement therapy (RRT), 4 by hemodialysis (HD) and 1 by peritoneal dialysis (PD).

### 3.1. Correlation of Different Methods

In the CPKD patients, Pearson correlation analysis showed all methods gave results that had statistical significance to the 24-h CrCl (*p* < 0.05, two-tailed). Correlation coefficients (r) was highest for CG 0.906 (r^2^ = 0.820), followed by CKD-EPI_crea/cys_ 0.901 (r^2^ = 0.812), CKD-EPI_cys_ 0.895 (r^2^ = 0.801) CKD-EPI_crea_ 0.863 (r^2^ = 0.744) and MDRD 0.799 (r^2^ = 0.638) (Table 1).

### 3.2. Comparison of Different Methods of eGFR by Stages and Creatinine Concentration

The CG and MDRD formulas had mean eGFR values of 58.37 and 59.42 mL/min respectively which were similar to the mean CrCL value of 59.37 mL/min. The CKD-EPI_cr_ had a value of 54.79 mL/min which borderline significant and the other equations had significant differences compared with CrCl (Table 2).

Table 2 also shows comparisons of eGFR in the five stages of CKD. The CG equation gave good agreement compared to 24-h CrCl in all but stage 5 CKD. The MDRD equation compared well in all except stage 4 as opposed to the CKD-EPI_cys_ formula which showed good agreement with CrCl in stage 4 only. Favorable agreement with CrCl was observed for CKD-EPI_crea_ results in stage 2, 3 and 5 and for the CKD-EPI_crea/cys_ in stages 4 and 5.

The majority of the patients fell within stage III for CrCl and the eGFR formula with the highest number was designated by CKD-EPI_cys_ and the lowest, CG (Table 3).

### 3.3. Comparison of Methods by Normal vs. Abnormal Creatinine Levels

The normal range for serum creatinine at the Chemical Pathology Laboratory is 9–124 μmol/L. In this study, serum creatinine among the participants ranged from 22–1417 μmol/L with mean 236.77 ± 247.29 μmol/L. Creatinine values of ≤124 μmol/L were observed for 53 subjects while 87 had values >124 μmol/L. Mean creatinine ≤124 μmol/L was 81.23 ± 28.11 μmol/L and the mean value >124 μmol/L was 331 ± 272.70 μmol/L. Comparisons of GFR were made using the paired t-test by separating the population into those with serum creatinine concentrations ≤124 μmol/L (*n* = 53) and >124 μmol/L (*n* = 87). In the former group results compared well with 24-h CrCl and were statistically insignificant for CG, MDRD and the CKD-EPI_crea_ equations while for the latter the CG and MDRD equations compared well (Table 4).

### 3.4. Comparison of Methods by Age and Gender

Table 5 shows comparison of the formulas in patients 60 years old and over and in the age group < 60 years while Table 6 shows the comparison of the same in males and females. The CG, MDRD and the CKD-EPI_crea_ equations performed well and had similar results compared with 24-h CrCl in patients 60 years and over, and females (Table 5 and Table 6). The MDRD and the CKD-EPI_crea_ equations also showed results that were similar to 24-h CrCl in patients less than 60 years (Table 5). The MDRD gave the most comparable result to 24-h CrCl in males (*p* = 0.12) (Table 6).

### 3.5. Comparison of Methods by Ranges of CrCl

In the sub-group of patients with CrCl < 60 mL/min, the CG formula proved to be similar compared with 24-h CrCl while the MDRD and CG formulas were comparable when CrCl ≥ 60 mL/min (Table 7). All methods underestimated GFR at CrCl ≥ 60 mL/min (Table 7).

### 3.6. Determination of Bias

**Figure 2:**
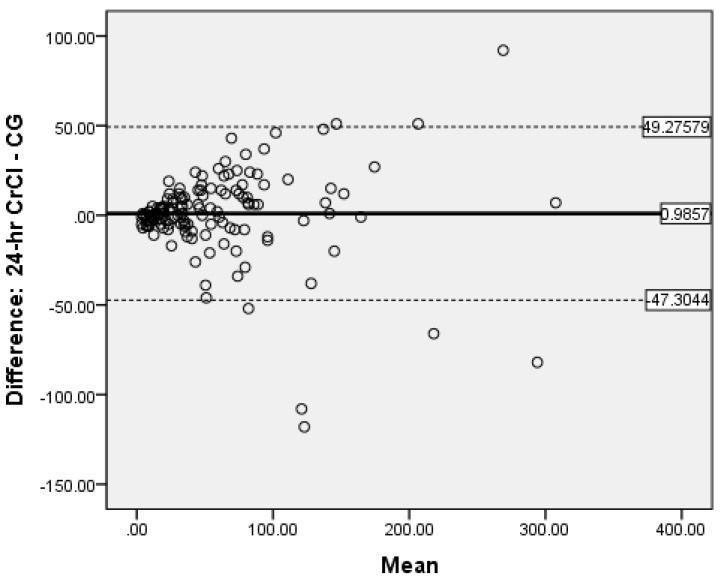
Bland-Altman plot of CG vs. 24-h CrCl.

**Figure 3:**
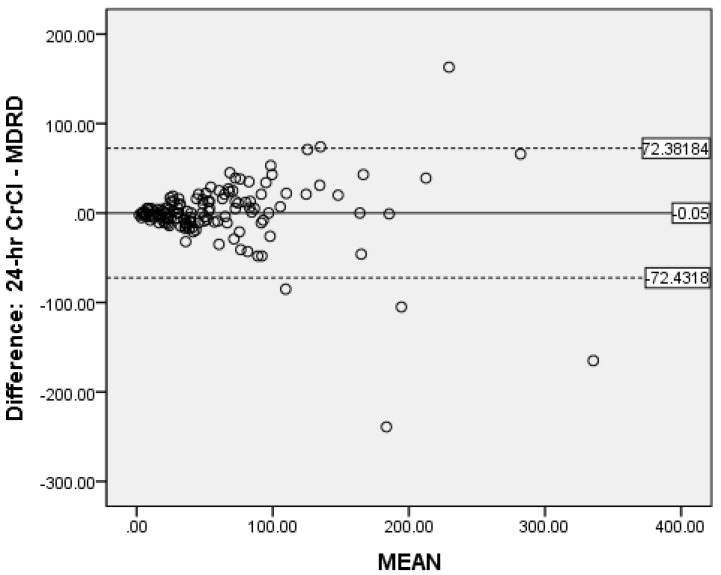
Bland-Altman plot of MDRD vs. CrCl.

**Figure 4:**
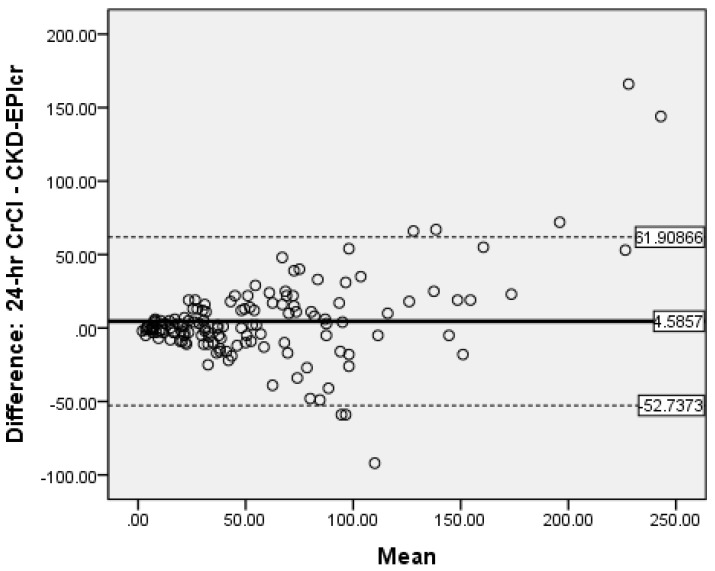
Bland-Altman plot of CKD-EPIcrea vs. CrCl.

Bland-Altman analysis was used to measure the accuracy of the different methods compared to 24-h CrCl. There was no proportional bias for CG (Figure 2) and MDRD (Figure 3), while for CKD-EPI_crea_ there was significant bias (Figure 4). The CG equation was positively biased (0.9857) with limits of agreement (LOA) of −47.3044 to 49.27579.

The MDRD had a negative bias (−0.05) with LOA of −72.4318 to 72.38184. The results for the CG and MDRD formulas were satisfactory, but unacceptable for the CKD-EPI_crea_ (bias 4.5857, LOA −52.7373 to 61.90866) equation. Precision was greater for the CG formula compared to 24-h CrCl. Bland-Altman analyses were not performed for the CKD-EPI_cys_, and CKD-EPI_crea/cys_ as the independent t-tests showed significant bias between the differences of the means.

## 4. Discussion

To our knowledge this is the first published report of the determination of eGFR using the CG, MDRD, CKD-EPI_crea_, CKD-EPI_cys_ and CKD-EPI_crea/cys_ equations and its comparison with 24-h CrCl in a Caribbean population mainly of African descent. In this study, results from Pearson correlation analysis indicated strong positive correlations for cystatin- and creatinine-based equations with 24-h CrCl, and these values were also highly correlated with each other. However, the correlations between serum creatinine and cystatin-based eGFR values were lower and moderately negative.

In our study, the CG formula showed the highest correlation (r = 0.906, *p* < 0.05) while the MDRD formula displayed weakest correlation (r = 0.799, *p* < 0.05). Results reported for CG and MDRD formulas in a study by Hahn et al. of patients before autologous and allogeneic bone marrow transplant showed similar order though weaker values as the correlation for the CG formula (r = 0.63) was slightly higher than the MDRD formula (r = 0.54) [37]. Moreover, findings from a cross-sectional study by Adebisi where 24-h CrCl was exposed to correlational analysis showed strong positive correlation with values of 0.905 and 0.904 for CG and MDRD formulas respectively [38]. Also, recent findings by Das et al. of a cross-sectional study of 100 patients with CKD, demonstrates a strong positive correlation between CrCl and CKD-EPI (r = 0.848, *p* < 0.001) and MDRD (r = 0.841, *p* < 0.001) respectively [39].

When the overall means were compared in this study, the eGFRs determined by CG, CKD-EPI_crea_, CKD-EPI_crea/cys_ and CKD-EPI_cys_ equations in decreasing order of values were lower than 24-h CrCl, while that produced by MDRD was slightly higher. Lower mean eGFR values for CG of 32.18 mL/min/1.73 m^2^, MDRD 26.56 mL/min/1.73 m^2^ and 24-h CrCl of 21.75 mL/min/1.73 m^2^ were found in 64 CKD patients in Nigeria with stable disease [38]. The differences in this study and ours could be due to more patients with advance CKD as well as dissimilar patient characteristics. Furthermore, our study demonstrated that the cystatin-based equations for patients with stage 1–4 CKD had lower eGFR values than creatinine-based equations. The CKD-EPI_crea/cys_ gave lower GFR estimates; findings that are similar to that obtained by Hu et al. [40].

In this study our findings of the MDRD formula producing a mean value that was greater than that obtained by 24-h CrCl and CG formula is in contrast to evidence of other researchers such as Verhave et al. who reported lower values. Also, Verhave et al. examined data of 8592 participants in the Prevention of Renal and Vascular End-stage Disease study and found that the mean GFRs for 24-h CrCl, MDRD and CG were in the range of 77.5–94.6 mL/min/1.73 m^2^, which were higher than ours. The disparity between the findings of Verhave and ours could be differences in weight, body mass index (BMI) and stage of CKD [41]. However, in a retrospective study of 91 participants, the mean GFRs for 24-h CrCl, MDRD (2006), EPI-(2009) and CG were in the range 53.34–57.21 mL/min/1.73 m^2^, which was closer to ours [42].

The majority of the patients in our study according to 24-h CrCl was in stage III. The finding is similar to that of Krzanowski et al. who reported that the highest proportion of CKD patients were designated to stage 3 [43]. A comparison of methods with 24-h CrCl revealed that CG, MDRD and CKD-EPI_crea_ equations overestimates in stage 2–5 and underestimates in stage 1. CG formula had the strongest association in stage 1–3 CKD, sub-population with CrCl < 60 mL/min and with both serum creatinine results ≤124 μmol/L and >124 μmol/L. Results of comparison of the MDRD formula with 24-h CrCl indicated superior performance in males, 24-h CrCl levels ≥ 60 mL/min/1.73 m^2^ and the sub-population < 60 years old. The CKD-EPI_crea_ equation had the strongest relationship with 24-h CrCl in stage 5 CKD, in the age-group > 60 years old and in females. CG tended to overestimates 24-h CrCl in the <60 age group and underestimates in the age group ≥60 years compared to the MDRD and CKD-EPI_crea_ formulas.

Kumar and colleagues who performed a retrospective study involving 91 CKD patients reported that the CG formula showed good approximation to 24-h CrCl only in stage 2–4 CKD while it was stage 2–5 for the MDRD (2006) formula [42]. Similar to our study, Hu et al. reported that the serum creatinine-based equations such as MDRD and CG showed better agreement with 24-h CrCl particularly for patients with stage 2–5 CKD [40]. They also indicated that the MDRD equation performed better for females or elderly patients [40]. In contrast, according to a meta-analyses by Zou et al., CKD-EPI_cys_ was the most accurate compared with CKD-EPI_crea/cys_, and CKD-EPI_crea_ and performed best in the analyses of sub-group such as those persons with mGFR < 60 mL/min/1.73 m^2^, aged less than 70 years and those participants who were of the Asia ethnic group [28].

Studies have highlighted the significance of careful calibration of serum creatinine measurements in order to accurately and reliably determine the 24-h CrCl or eGFR in patients with normal or slightly decreased kidney function [44,45]. The calibrated Roche enzymatic assay utilized in our study is traceable to reference IDMS and therefore provide accurate results for 24-h CrCl as well as CG, MDRD and CKD-EPI_crea_ [46].

The evaluation of bias, a measure of systematic error in this observational study of validated CKD patients by Bland-Altman analysis showed satisfactory negligible negative bias (−0.05) for the MDRD formula (LOA of −72.4318 to −72.3318) and a very small positive bias (0.9857) for CG (LOA of −47.3044 to −49.27579). This indicate that when the entire study population was considered, the MDRD equation underestimates 24-h CrCl by 0.05 mL/min and the CG formula overestimates the same by 0.99 mL/min. This shows good global agreement between 24-h CrCl and MDRD as well as CG. Notably, in our study the measure of precision was reported as the 95% limits of agreement and the CG formula was more precise than the MDRD equation. Fairly similar findings were reported by Froissart et al. in a large adult European population where the MDRD and CG formulas overestimated measured GFR (renal clearance of ^51^Cr-EDTA) by 0.99 mL/min/1.73 m^2^ and 1.94 mL/min/1.73 m^2^ respectively [22]. In the African-American Study of Hypertension and Kidney Disease with 1703 participants, the CG formula overestimates the measured GFR ^125^-I-iothalamate GFR by 2.7 mL/min/1.73 m^2^ [47]. The reasons for the inconsistency in eGFR values are unclear, but it may be due to discrepancies in patient characteristics.

In our study, there was significant positive bias with CKD-EPI_crea_ as this equation overestimates 24-h CrCl by 4.59 mL/min. Also, Bland-Altman analyses were not performed for the CKD-EPI_cys_ and the CKD-EPI_crea/cys_ as the independent t-tests showed significant bias between the differences of the means and 24-h CrCl. In contrast, in a meta-analysis of 35 studies with 23,667 persons, eGFR measured using CKD-EPI_cys_ had less bias compared with CKD-EPI_crea/cys_, and CKD-EPI_crea_. The authors also reported that the projected values from CKD-EPI_crea/cys_ displayed greater accuracy than those from CKD-EPI_crea_ [28]. Moreover, a systematic and meta-analysis 48 studies of 26,875 patients from diverse populations showed that eGFR values using CKD-EPI equations had lower mean bias and performed better than the MDRD formula [48]. Also, in an earlier meta-analyses involving 1,130,472 adults from 25 different patient population, CKD-EPI equations had less bias and greater accuracy than the MDRD formula [49].

To our knowledge, cystatin C has not been measured prior to this study in Jamaica. The two cystatin C based formulas were positive and fairly correlated with 24-h CrCl, CG, and MDRD and CKD-EPI_cr_ equations. However, they mostly overestimates 24-h CrCl and could not accurately categorized the CKD patients in stage 1–5. This means that the cystatin C-based formulas used in this study may unlikely be the best estimators of GFR in Caribbean patients with African decent as observed in Jamaica. Bukabau et al. reported results of a cross-sectional study of 494 adults from two Sub-Saharan African populations where CKD-EPI_crea/cys_ and CKD-EPI_cys_ had similar performance, but neither CKD-EPI_crea/cys_ nor CKD-EPI_cys_ significantly improve eGFR compared with CKD-EPI_crea_ when assessed against iohexol as the reference standard [50]. However, in a large diverse population study, CKD-EPI_crea/cys_ performed better than either CKD-EPI_cys_ or CKD-EPI_crea_ and may be valuable in confirming chronic renal disease [51]. Nevertheless, the slightly enhanced performance of CKD-EPI_crea/cys_ or CKD-EPI_cys_ may not be adequately enough to merit the added cost of using cystatin C in daily practice coupled with the longer turnaround time [25].

There were several limitations in this study. First, a gold standard like the clearance of inulin or ^125^-I-iothalamate would be the best method to evaluate GFR and make comparisons to determine which formula gives the most accurate eGFR. However, with limited resources the most commonly used test, the 24-h CrCl was utilized. Correlations of eGFR to CrCl were similar to those seen in other studies.

For patients with CKD, the accuracy of 24-h CrCl is also affected by the tubular secretion from the peritubular capillaries into the lumen or into the gastrointestinal tract, and error in serum creatinine levels due to the reaction of chromogens such as ascorbic acid and acetone (based on the Jaffe reaction) [14,52]. However, it should be noted that the Jaffe assay used to determine creatinine in this study is traceable to IDMS which improve the accuracy of the results [53].

The authors also noted that the overall performance of the formulas used in this study could be affected by different parameters such as all are influenced by age, CG by BMI and body weight, and CKD-EPI and MDRD do not consider the lean body mass and weight of patient. These factors could introduce overall bias in the study [54]. However, given that the study is conducted on persons of mainly African descent in Jamaica, there are less variations in anthropometric parameters.

Furthermore, another limitations is that most of the CKD patients recruited in the study were deemed to be in stage 3 by the 24-h CrCl. We are aware that this could introduce bias and we attempted to mitigate this possibility by one of the authors, a nephrologist ensuring that the clinical characteristics, creatinine levels and clearance were associated with stage 3 disease.

Our population was totally Afro-Caribbean and extensive studies have not been done in this population. Populations in most studies have proportionately less black subjects and differences have even been shown in South African vs. and blacks in United States of America and European [14].

The differences in eGFR estimates between the cystatin and creatinine-based equations warrants the need for population-based studies in Jamaica with satisfactory sample sizes to validate these eGFR formulas. This will afford the necessary data required to determine the most suitable eGFR equation to utilize in assessing CPD patients in the local setting.

## 5. Conclusions

The CG formula compared most favorably and is the best alternative to 24-h CrCl given the limitations of the latter. The performance of 24-h CrCl was enhanced as the creatinine assay used in this study is traceable to IDMS and stringent measures were employed regarding urine collection. It could be recommended as a substitute to provide the best estimate of GFR in our population. The MDRD formula should be used with caution as there is a tendency to overestimate GFR. More studies are warranted to verify the role of cystatin C in predicting development of CKD as well as other adverse outcomes associated with the disease. Studies comparing estimated GFR to measured GFR using exogenous markers such as inulin or iohexol would be best to determine the method most appropriate for our population as 24-h CrCl has a number of limitations including its overestimation of the true GFR.

## Figures and Tables

**Table 1:** Correlation of methods of eGFR and clinical variables.

		CrCl	CG	MDRD	CKD-EPIcr	CKD-EPIcys	CKD-EPIcr-Cys	Creatinine	Cystatin C	Urea
N		140	140	140	140	140	140	140	140	140
CrCl	Pearson’ coefficient p (Sig. 2-tailed)	1	0.906 < 0.05	0.799 < 0.05	0.863 < 0.05	0.895 < 0.05	0.901 < 0.05	−0.511 < 0.05	−0.623 < 0.05	−0.600 < 0.05
CG	Pearson’ coefficient p (Sig. 2-tailed)	0.906 < 0.05	1 < 0.05	0.899 < 0.05	0.915 < 0.05	0.869 < 0.05	0.921 < 0.05	−0.502 < 0.05	−0.588 < 0.05	−0.593 < 0.05
MDRD	Pearson’s coefficient p (Sig. 2-tailed)	0.799 < 0.05	0.899 < 0.05	1 < 0.05	0.929 < 0.05	0.840 < 0.05	0.921 < 0.05	−0.514 < 0.05	−0.591 < 0.05	−0.585 < 0.05
CKD-EPIcr	Pearson’s coefficient p (Sig. 2-tailed)	0.863 < 0.05	0.915 < 0.05	0.929 < 0.05	1 < 0.05	0.902 < 0.05	0.972 < 0.05	−0.622 < 0.05	−0.699 < 0.05	−0.697 < 0.05
CKD-EPIcys	Pearson’ coefficient p (Sig. 2-tailed)	0.895 < 0.05	0.869 < 0.05	0.840 < 0.05	0.902 < 0.05	1 < 0.05	0.975 < 0.05	−0.565 < 0.05	−0.753 < 0.05	−0.691 < 0.05
CKD-EPIcr-cys Pearson’s coefficient *p* (Sig. 2-tailed)		0.901 < 0.05	0.921 < 0.05	0.921 < 0.05	0.972 < 0.05	0.975 < 0.05	1 < 0.05	−0.599 <0.05	−0.735 < 0.05	−0.701 < 0.05
Creatinine	Pearson’s coefficient *p* (Sig. 2-tailed)	−0.511 < 0.05	−0.502 < 0.05	−0.514 < 0.05	−0.622 < 0.05	−0.565 < 0.05	−0.599 < 0.05	1 < 0.05	0.838 < 0.05	0.782 < 0.05
Cystatin C	Pearson’s coefficient *p* (Sig. 2-tailed)	−0.623 < 0.05	−0.588 < 0.05	−0.591 < 0.05	−0.699 < 0.05	−0.753 < 0.05	−0.735 < 0.05	0.838 < 0.05	1 < 0.05	0.829 < 0.05
Urea	Pearson’s coefficient *p* (Sig. 2-tailed)	−0.600 < 0.05	−0.593 < 0.05	−0.585 < 0.05	−0.697 < 0.05	−0.691 < 0.05	−0.701 < 0.05	0.782 < 0.05	0.829 < 0.05	1

**Table 2:** Comparison of CrCl and eGFR by method (mL/min).

Stages CKD	Population (n)	24-h Creat Cl	CG	*p*	MDRD	*p*	CKD-EPI Crea	*p*	CKD-EPI Cys	*p*	CKD-EPI Crea/Cys	*p*
V	22	7.32	9.50	<0.05	8.64	0.14	7.82	0.53	11.5	<0.05	8.95	0.07
IV	24	20.67	22.83	0.34	26.63	<0.05	24.58	<0.05	21.29	0.63	22.21	0.27
III	41	41.10	41.95	0.74	47.51	0.26	43.46	0.34	29.17	<0.05	34.22	<0.05
II	27	74.33	76.78	0.72	85.04	0.31	80.70	0.30	47.63	<0.05	60.85	<0.05
I	26	152.42	139.38	0.08	130.85	0.08	113.35	<0.05	76.96	<0.05	94.08	<0.05
ALL	140	59.37	58.39	0.64	59.42	0.99	54.79	0.07	37.48	<0.05	44.44	<0.05

**Table 3:** The number of patients at different stage CKD by method (mL/min).

Stage	CrCl	CG	MDRD	CKD-EPI Crea	CKD-EPI Cys	CKD-EPI Crea-Cys
I	26	24	26	29	9	16
II	27	26	20	17	13	21
III	41	38	46	44	49	45
IV	24	28	27	26	44	32
V	22	24	21	24	25	26
All	140	140	140	140	140	140

**Table 4:** Comparison of methods for serum creatinine ≤ 124 μmol/L and creatinine > 124 μmol/L.

Serum Creatinine ≤ 124 μmol/L					
Method	N	Mean	SD	SE	*p*
24-h Creat Cl	53	106.26	64.38	8.84	-
Cockcroft-Gault	53	105.72	65.27	8.97	0.92
MDRD	53	109.79	69.48	9.54	0.66
CKD-EPIcrea	53	99.11	37.19	5.11	0.25
CKD-EPIcys	53	61.02	26.88	3.69	<0.05
CKD-EPIcre/cys	53	77.47	32.29	4.44	<0.05
Serum Creatinine > 124 μmol/L					
24-h Creat Cl	87	30.80	22.96	2.46	-
Cockcroft-Gault	87	29.55	20.03	2.15	0.32
MDRD	87	28.74	16.48	1.77	0.16
CKD-EPIcr	87	27.78	16.76	1.80	<0.05
CKD-EPIcys	87	23.14	11.90	1.28	<0.05
CKD-EPIcrea/cys	87	24.32	13.45	1.44	<0.05

**Table 5:** Comparison of methods (mL/min) in patients <60 years of age vs. patients 60 years and over.

<60 years					
Method	N	Mean	SD	SE	*p*
24-h CrCl	73	79.96	62.77	7.35	-
Cockcroft-Gault	73	83.33	67.24	7.87	0.36
MDRD	73	78.52	73.56	8.61	0.80
CKD-EPIcrea	73	71.37	49.39	5.78	0.05
CKD-EPIcys	73	46.89	30.79	3.60	<0.05
CKD-EPIcrea/cys	73	57.15	40.00	4.68	<0.05
60 years and over					
24-h CrCl	67	36.94	25.85	3.40	-
CG	67	31.21	20.08	2.45	<0.05
MDRD	67	38.61	26.28	3.21	0.40
CKD-EPIcrea	67	36.72	26.43	3.23	0.91
CKD-EPIcys	67	27.22	15.20	1.86	<0.05
CKD-EPIcrea/cys	67	30.60	18.65	2.28	<0.05

**Table 6:** Comparison of methods (mL/min) by gender.

Males					
Method	N	Mean	SD	SE	*p*
24-h CrCl	65	60.42	54.15	6.72	-
Cockcroft-Gault	65	52.57	47.29	5.74	< 0.05
MDRD	65	53.92	41.57	5.16	0.12
CKD-EPIcrea	65	51.63	35.76	4.44	<0.05
CKD-EPIcys	65	37.34	24.46	3.03	<0.05
CKD-EPIcrea/cys	65	42.82	28.96	3.59	<0.05
Females					
24-h CrCl	75	58.47	59.34	6.85	-
Cockcroft-Gault	75	63.43	64.29	7.42	0.13
MDRD	75	64.19	71.33	8.24	0.21
CKD-EPIcrea	75	57.52	49.40	5.71	0.77
CKD-EPIcys	75	37.60	28.17	3.25	<0.05
CKD-EPIcr-cys	75	45.85	38.34	4.43	<0.05

**Table 7:** Comparison of methods for 24-h CrCl < 60 mL/min vs. 24-h CrCl ≥ 60 mL/min.

CrCl < 60 mL/min					
Method	N	Mean	SD	SE	*p*
24-h CrCl	87	26.92	16.12	1.73	-
Cockcroft-Gault	87	28.47	19.77	2.12	0.26
MDRD	87	30.13	19.47	2.09	<0.05
CKD-EPIcrea	87	29.24	20.22	2.17	0.07
CKD-EPIcys	87	22.53	11.18	1.20	<0.05
CKD-EPIcr/cys	87	24.52	14.14	1.52	<0.05
CrCl ≥ 60 mL/min					
24-h CrCl	53	112.64	59.49	8.17	-
Cockcroft-Gault	53	107.49	63.22	8.68	0.31
MDRD	53	107.51	70.84	9.73	0.52
CKD-EPIcrea	53	96.72	38.87	5.34	<0.05
CKD-EPIcys	53	62.02	25.91	3.56	<0.05
CKD-EPIcr/cys	53	77.15	32.33	4.44	<0.05

## Data Availability

The conditions of our ethics approval does not permit public archiving of the data supporting the conclusions of the study. However, data described in the manuscript, code book, and analytic code will be made available upon request.
